# Use of long non-coding RNAs for the molecular diagnosis of papillary thyroid cancer

**DOI:** 10.3389/fonc.2022.924409

**Published:** 2022-09-05

**Authors:** Daham Kim, Juyeon Yu, Jiwon Kim, Yoon-a Hwang, Jin Kyong Kim, Cheol Ryong Ku, Jung Hyun Yoon, Jin Young Kwak, Kee-Hyun Nam, Eun Jig Lee

**Affiliations:** ^1^ Department of Internal Medicine, Institute of Endocrine Research, Yonsei University College of Medicine, Seoul, South Korea; ^2^ Department of Surgery, Yonsei University College of Medicine, Seoul, South Korea; ^3^ Department of Radiology, Yonsei University College of Medicine, Seoul, South Korea

**Keywords:** long non-coding RNA, diagnosis, thyroid cancer, thyroid nodule, molecular test

## Abstract

**Objective:**

Improved molecular testing for common somatic mutations and the identification of mRNA and microRNA expression classifiers are promising approaches for the diagnosis of thyroid nodules. However, there is a need to improve the diagnostic accuracy of such tests for identifying thyroid cancer. Recent findings have revealed a crucial role of long non-coding RNAs (lncRNAs) in gene modulation. Thus, we aimed to evaluate the diagnostic value of selected lncRNAs from The Atlas of Noncoding RNAs in Cancer (TANRIC) thyroid cancer dataset.

**Methods:**

LncRNAs in TANRIC thyroid cancer dataset that have significantly increased or decreased expression in papillary thyroid cancer (PTC) tissues were selected as candidates for PTC diagnosis. Surgical specimens from patients who underwent thyroidectomy were used to determine the separation capability of candidate lncRNAs between malignant and benign nodules. Fine needle aspiration samples were obtained and screened for candidate lncRNAs to verify their diagnostic value.

**Results:**

LRRC52-AS1, LINC02471, LINC02082, UNC5B-AS1, LINC02408, MPPED2-AS1, LNCNEF, LOC642484, ATP6V0E2-AS1, and LOC100129129 were selected as the candidate lncRNAs. LRRC52-AS1, LINC02082, UNC5B-AS1, MPPED2-AS1, LNCNEF, and LOC100129129 expression levels were significantly increased or decreased in malignant nodules compared to those in benign nodules and paired normal thyroid tissues. The combination of LRRC52-AS1, LINC02082, and UNC5B-AS1 showed favorable results for the diagnosis of PTC from fine needle aspirates, with 88.9% sensitivity and 100.0% specificity.

**Conclusions:**

LncRNA expression analysis is a promising approach for advancing the molecular diagnosis of PTC. Further studies are needed to identify lncRNAs of additional diagnostic value.

## Introduction

Thyroid nodules are common and clinically significant because about 5% of thyroid nodules are malignant (1). The incidence of thyroid cancer is on the rise and is expected to become the fourth most common cancer globally (2). The distinction between benign and malignant thyroid nodules has important therapeutic implications. Fine needle aspiration (FNA) cytology is the gold standard for the diagnosis of thyroid nodules. However, its results indicate cytologically indeterminate nodules (Bethesda classification III-IV) in approximately 20% of cases, posing a diagnostic challenge (3).

Approaches for obtaining information on cancer risk are necessary for establishing management strategies. With the recent development of molecular biological research and genetic analysis technology, molecular tests for the accurate diagnosis of thyroid cancer are being developed rapidly (4, 5). Improved molecular testing for common somatic mutations and identification of mRNA and microRNA expression classifiers have emerged as the most promising approaches (6). Somatic mutation testing is currently the most studied molecular diagnostic test for FNA biopsy (7, 8). However, mutation tests, except for the BRAF mutation, are not yet widely available in the real clinical world, and mutations alone cannot explain all aspects of the tumor. The analysis of differentially expressed genes has emerged as an alternative to mutation testing (9). However, although up to 70% of the human genome is transcribed to RNA, only 2% of it represents protein-coding genes, and transcript levels are not sufficient for the prediction of protein levels (10, 11). MicroRNAs act by base-pairing with target mRNA to negatively regulate the latter’s expression; recently, microRNA analysis has gained an important place in the study of molecular markers (12–14).

The Encyclopedia of DNA Elements project revealed that approximately 80% of the human genome is transcribed into 14,880 long non-coding RNAs (lncRNAs) from 9,277 loci (15). LncRNAs are defined as non-protein coding transcripts longer than 200 nucleotides, although recent literature suggesting some lncRNAs can encode small peptides or small proteins (16). Currently, lncRNA research has moved to the forefront of human cancer research, as recent findings have revealed a crucial role for lncRNAs in gene modulation. LncRNAs are primarily involved in the epigenetic regulation of the expression of various genes at different levels, including chromatin, splicing, transcription, and the post-transcription stages (17–19). LncRNAs are potent biomarkers, although their expression levels are considerably lower than those of mRNAs. Because lncRNAs do not encode proteins and perform their biological functions directly, most of their functions correlate with their expression levels (15, 17). The expression patterns of lncRNAs are more tissue-specific than those of protein-coding genes, and this property makes them useful as highly specific diagnostic biomarkers (20).

The aims of this study were to evaluate the diagnostic value of selected lncRNAs from our previous study and The Atlas of Noncoding RNAs in Cancer (TANRIC) thyroid cancer dataset to determine whether lncRNA expression analysis can be a promising approach for advancing the molecular diagnosis of papillary thyroid cancer (PTC) (21, 22).

## Materials and methods

### TANRIC thyroid cancer dataset

We obtained the data of lncRNAs, expressed in reads per kilobase per million (RPKM), of 59 paired PTC tissues and normal thyroid tissues from TANRIC thyroid cancer dataset, which characterizes the expression profiles of lncRNAs in The Cancer Genome Atlas (TCGA) PTC data sources (22–24). The data were used for the selection of lncRNAs as candidates for PTC diagnosis.

### Patients and samples

Surgical specimens were obtained from patients who underwent thyroidectomy, and FNA samples were obtained from patients who underwent FNA biopsies of thyroid nodules between June 2019 and August 2021 at the Yonsei Cancer Center (Seoul, South Korea). All samples were immediately stored in RNAlater (Ambion, Austin, TX, USA) and subsequently stored at −80°C until use. Since there is no benign nodule in TANRIC thyroid cancer dataset, surgical specimens were used to determine the separation capability of candidate lncRNAs between malignant and benign nodules. FNA samples were used to verify the candidate lncRNAs and evaluate their diagnostic performance. Clinicopathological information was retrospectively collected from databases at our institution. This study was conducted in accordance with the 1964 Declaration of Helsinki and approved by the Institutional Review Board of Severance Hospital (No. 4-2019-0335). Informed consent was obtained from all the patients.

### RNA isolation and real-time PCR analysis

RNA from surgical specimens or FNA samples was extracted using the RNeasy Plus Micro Kit (Qiagen, Valencia, CA, USA). To confirm the diagnostic potential of the lncRNAs in surgical specimens, we synthesized cDNA from 1 μg of total RNA using the QuantiTect Reverse Transcription Kit (Qiagen). Primer sequences are shown in **Table 1**. Real-time PCR mixtures consisted of 10 μL Power SYBR^®^ Green PCR Master Mix (Applied Biosystems, Foster City, CA, USA), 5 pmol each of forward and reverse primers, 50 ng of diluted cDNA template, and sterile distilled water to a final volume of 20 μL. PCR was performed on an ABI StepOnePlus Real-Time PCR system (Applied Biosystems) (21). All reactions were performed in duplicate. GAPDH was used as an internal control.

**Table 1:** Primer sequences used for real-time PCR.

Gene	Forward 5′-3′	Reverse 5′-3′
LRRC52-AS1	ATAAGGGGATCTGCAAGGCA	AACAGGTTCCTTCAACCAGGG
LINC02471	ATCCCTTGGCATATGGTGTGTT	ACTCAGGATATGGAGTTGCGA
LINC02082	AGAAACCTTCTGCCACCCAAA	GCTGAACGCCCAATACAGGA
UNC5B-AS1	ACAAGCCTGCCTTCTTGGAG	GTGGCGCTTGATTGGAACTC
LINC02408	GCTGTGTGATCCTAGATGGCT	TACATCCAGTGAGCAGGCAC
MPPED2-AS1	AGTTGCAGTCGTTCACCAGT	AGCAGCTCCAGGCATCAAG
LNCNEF	TGAGGAGCTGTTTGGGCAAT	TTGCGGATTCCACTCCCATC
LOC642484	GGACAGCAACCAGACCTGAG	ACAGCATGCACCTGCAACTA
ATP6V0E2-AS1	CCTTGACTCCTTGCGTCAGT	ACATCTTCCAGTCACGCTCC
LOC100129129	GTCTTGCTGTTTAGCGGCTC	GAAGCTGAAGAAAACGGGGC
GAPDH	GGAGCGAGATCCCTCCAAAAT	GGCTGTTGTCATACTTCTCATGG

For the analysis of FNA samples, the following TaqMan Assay Mixes were used: LRRC52-AS1 (Hs01594821_m1; JUN-QSY), LINC02082 (Hs00415625_m1; FAM-MGB), UNC5B-AS1 (Hs04274416_g1; ABY-QSY), and GAPDH (Hs02786624_g1; VIC-MGB). The real-time PCR mixtures consisted of 5 μL TaqPath 1-Step Multiplex Master Mix (Applied Biosystems), 1 μL each of TaqMan Assay Mix, 50 ng RNA sample, and sterile distilled water to a final volume of 20 μL. PCR was performed on an ABI QuantStudio 5 Real-Time PCR system (Applied Biosystems) according to the manufacturer’s instructions.

### Statistical analysis

IBM SPSS Statistics version 25 (IBM, Armonk, NY, USA) was used for all statistical analyses. Categorical variables are presented as the number and percentage, and continuous variables are presented as mean ± standard deviation. Continuous variables were compared using Student’s t-test for TANRIC thyroid cancer dataset, whereas the Mann–Whitney U and Kruskal–Wallis tests were performed using the patient data from our hospital. Categorical variables were compared using Fisher’s exact test. Differences with a p-value < 0.05 were considered statistically significant.

## Results

### Candidate lncRNA selection from TANRIC thyroid cancer dataset

**Figure 1:**
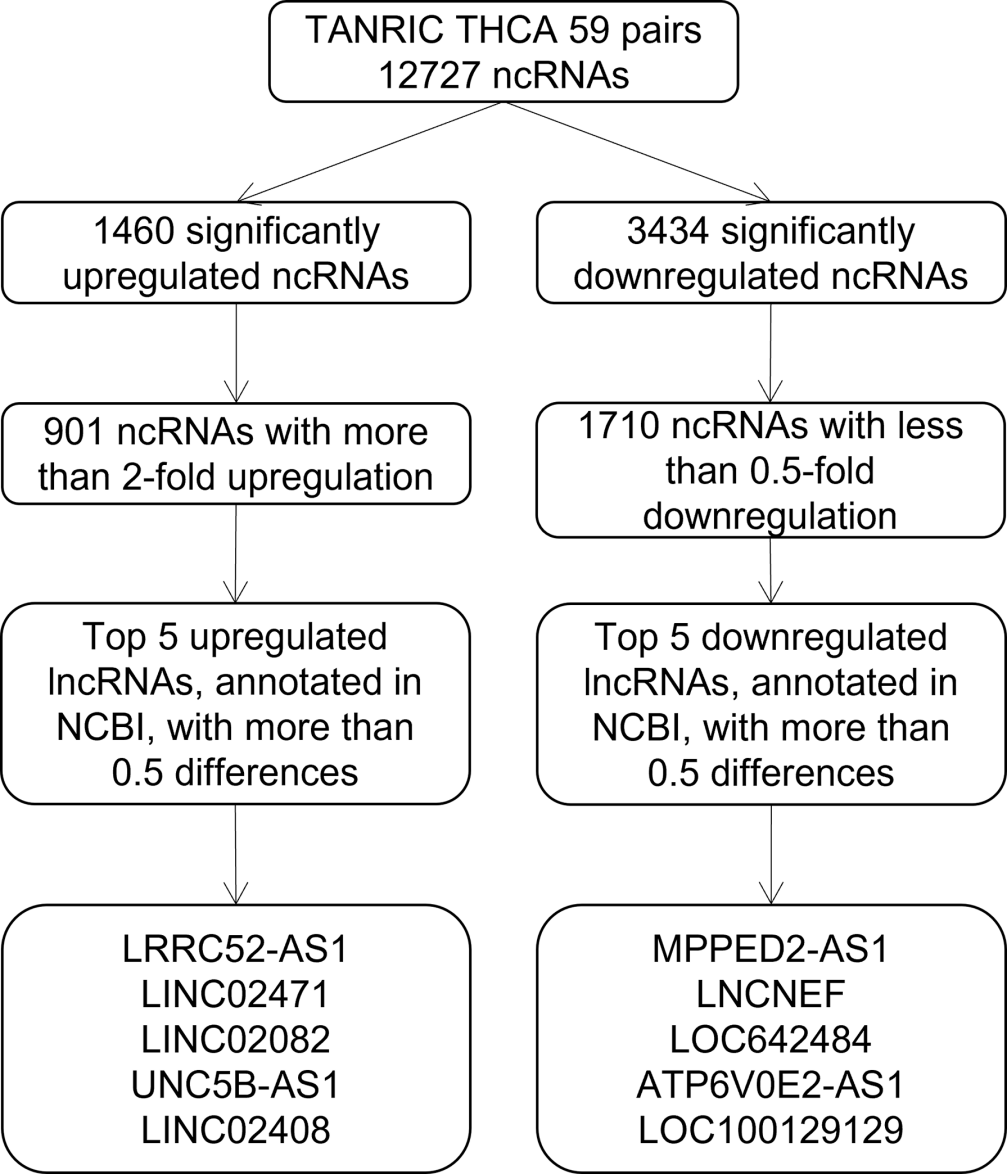
Flowchart for the selection of long non-coding RNAs (lncRNAs) as candidates for papillary thyroid cancer (PTC) diagnosis from The Atlas of Noncoding RNAs in Cancer (TANRIC) thyroid cancer dataset.

To identify candidate lncRNAs with diagnostic potential, we first compared the expression levels of 12,727 ncRNAs from 59 paired PTC tissues and normal thyroid tissues drawn from TANRIC thyroid cancer dataset. We selected 901 ncRNAs that were significantly (p < 0.05) upregulated (more than 2-fold) in PTC tissues and 1,710 ncRNAs that were significantly (p < 0.05) downregulated (less than 0.5-fold) in PTC tissues. Among them, we selected the top five upregulated and downregulated lncRNAs in the PTC tissues that were annotated in the National Center for Biotechnology Information (validated lncRNAs) with a value that differed by more than 0.5 between the PTC tissue and the paired normal thyroid tissue. The top five upregulated candidates for PTC diagnosis were LRRC52-AS1, LINC02471, LINC02082, UNC5B-AS1, and LINC02408, and the downregulated ones were MPPED2-AS1, LNCNEF, LOC642484, ATP6V0E2-AS1, and LOC100129129 (**Figure 1** and **Table 2**).

**Table 2:** Selected candidate long non-coding RNAs (lncRNAs) from The Atlas of Noncoding RNAs in Cancer (TANRIC) thyroid cancer dataset.

Official symbol	Gene ID	P-value	Mean N59	Mean T59	T59-N59	T59/N59
LRRC52-AS1	ENSG00000237463.1	9.58E−10	0.016932	2.76254	2.745608	163.1579
LINC02471	ENSG00000223914.1	8.23E−15	0.250378	10.0865	9.836122	40.28509
LINC02082	ENSG00000242268.2	1.32E−05	0.016668	0.659175	0.642507	39.54662
UNC5B-AS1	ENSG00000237512.2	6.17E−06	0.035556	1.38515	1.349594	38.95697
LINC02408	ENSG00000203585.3	2.11E−06	0.020193	0.666412	0.646219	33.00213
MPPED2-AS1	ENSG00000254489.1	1.25E−21	1.42859	0.153562	−1.27503	0.107492
LNCNEF	ENSG00000237396.1	3.20E−09	1.7388	0.201443	−1.53736	0.115852
LOC642484	ENSG00000206129.3	3.83E−16	0.663489	0.11583	−0.54766	0.174577
ATP6V0E2-AS1	ENSG00000204934.6	1.14E−06	3.28217	0.785087	−2.49708	0.239198
LOC100129129	ENSG00000255020.1	6.71E−13	4.23287	1.21616	−3.01671	0.287313

### Confirmation of diagnostic potential in surgical specimens

**Figure 2:**
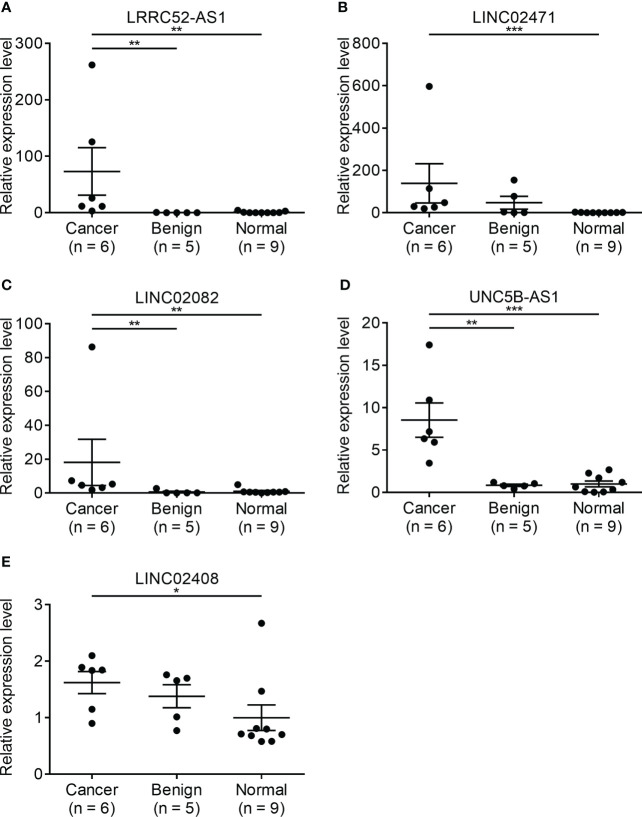
Expression levels of candidate long non-coding RNAs (lncRNAs) showing increased expression in surgical specimens at our institution. **(A)** LRRC52-AS1, **(B)** LINC02471, **(C)** LINC02082, **(D)** UNC5B-AS1, and **(E)** LINC02408. *p < 0.05, **p < 0.01, and ***p < 0.001.

**Figure 3:**
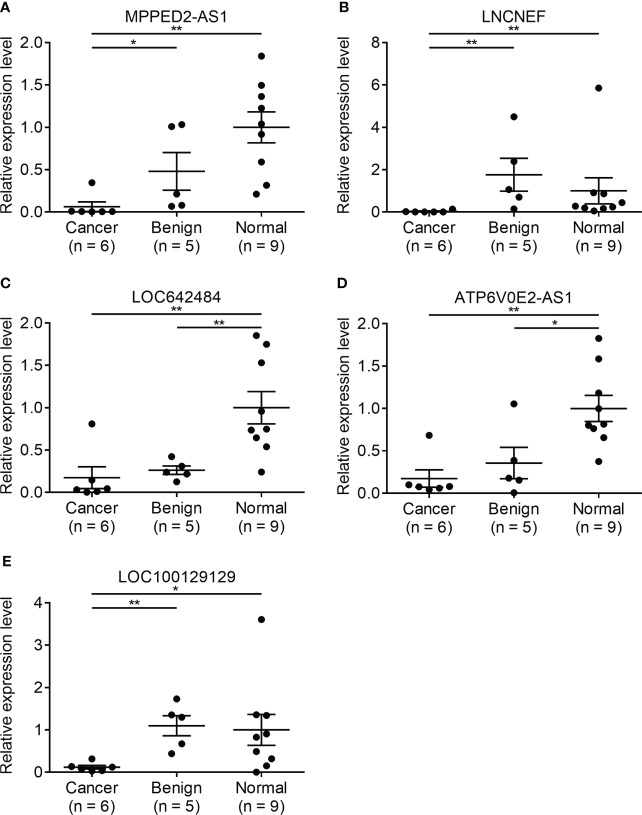
Expression levels of candidate long non-coding RNAs (lncRNAs) showing decreased expression in surgical specimens at our institution. **(A)** MPPED2-AS1, **(B)** LNCNEF, **(C)** LOC642484, **(D)** ATP6V0E2-AS1, and **(E)** LOC100129129. *p < 0.05 and **p < 0.01.

Since there is no benign nodule in TANRIC thyroid cancer dataset, expression levels of the candidate lncRNAs were confirmed in six malignant nodular (PTC) tissues, five benign nodular tissues, and nine paired normal thyroid tissues from nine patients with thyroid nodules who underwent thyroidectomy at our institution, before the application of candidate lncRNAs to FNA samples. The expression levels of LRRC52-AS1, LINC02082, and UNC5B-AS1 were significantly increased in the malignant nodules compared to those in the benign nodules and paired normal thyroid tissues (**Figure 2**). The expression levels of MPPED2-AS1, LNCNEF, and LOC100129129 were significantly reduced in the malignant nodules compared to those in the benign nodules and paired normal thyroid tissues (**Figure 3**). The expression levels of LINC02471, LINC02408, LOC642484, and ATP6V0E2-AS1 were significantly different between the malignant nodules and paired normal thyroid tissues as in TANRIC thyroid cancer dataset. However, they were not significantly different between the malignant and benign nodules. This indicates that the measurement of the expression levels of LRRC52-AS1, LINC02082, UNC5B-AS1, MPPED2-AS1, LNCNEF, and LOC100129129 could be used for differentiating between benign and malignant tumors in thyroid nodules. These lncRNAs were selected for further analysis.

### Receiver operating characteristic curves of candidate lncRNAs in TANRIC thyroid cancer dataset

**Figure 4:**
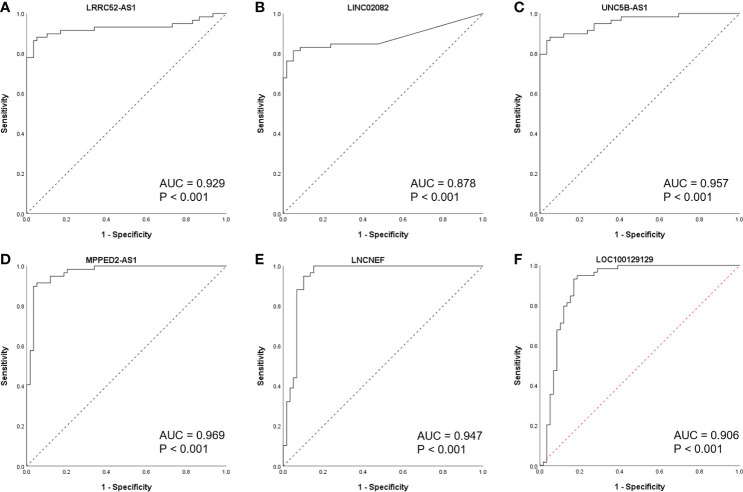
Receiver operating characteristic (ROC) curves of candidate long non-coding RNAs (lncRNAs) in The Atlas of Noncoding RNAs in Cancer (TANRIC) thyroid cancer dataset. **(A)** LRRC52-AS1, **(B)** LINC02082, **(C)** UNC5B-AS1, **(D)** MPPED2-AS1, **(E)** LNCNEF, **(F)** LOC100129129. AUC, area under the receiver operating characteristic (ROC) curve.

To further study the diagnostic potential of LRRC52-AS1, LINC02082, UNC5B-AS1, MPPED2-AS1, LNCNEF, and LOC100129129, the receiver operating characteristic (ROC) curves were plotted to analyze the potential diagnostic efficacy of these lncRNAs in TANRIC thyroid cancer dataset. These 6 lncRNAs, which all had AUC (area under the ROC curve) values greater than 0.878, showed potential for diagnostic use (**Figure 4**).

**Figure 5:**
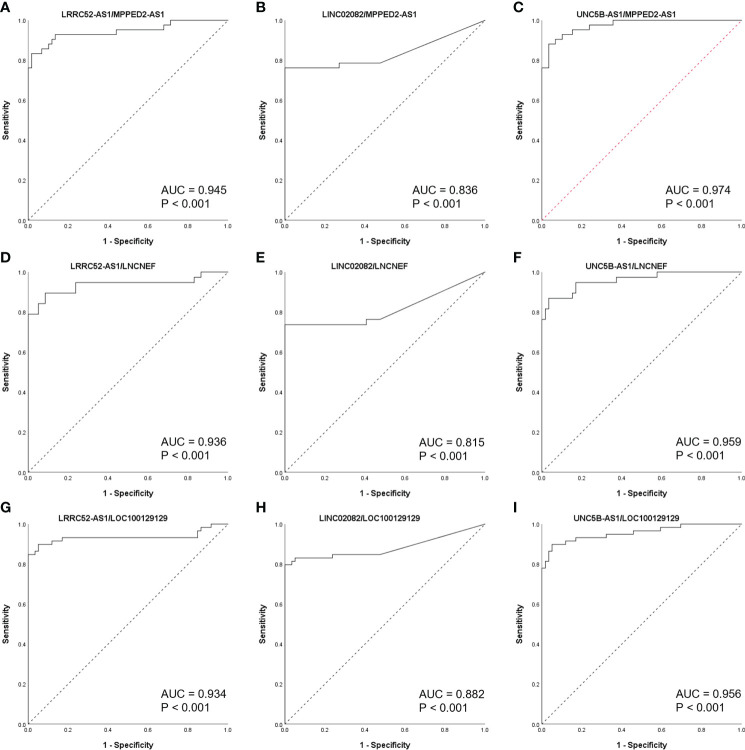
Receiver operating characteristic (ROC) curves of long non-coding RNA (lncRNA) combinations (calculated by dividing the increased lncRNA level by the decreased lncRNA level) in The Atlas of Noncoding RNAs in Cancer (TANRIC) thyroid cancer dataset. **(A)** LRRC52-AS1/MPPED2-AS1, **(B)**LINC02082/MPPED2-AS1, **(C)** UNC5B-AS1/MPPED2-AS1, **(D)** LRRC52-AS1/LNCNEF, **(E)** LINC02082/LNCNEF, **(F)** UNC5B-AS1/LNCNEF, **(G)** LRRC52-AS1/LOC100129129, **(H)** LINC02082/LOC100129129, and **(I)** UNC5B-AS1/LOC100129129. AUC, area under the ROC curve.

When the levels of lncRNAs with increased expression in malignant nodules were divided by those of lncRNAs with decreased expression in malignant nodules, the AUC values remained very high (**Figure 5**). These results indicate the possibility of using lncRNA expression levels as a factor in molecular tests of PTC.

### Application to FNA and verification

**Figure 6:**
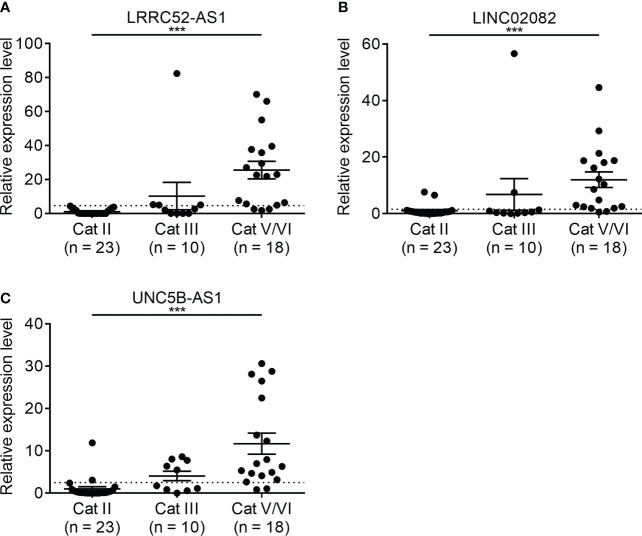
Expression levels of candidate long non-coding RNAs (lncRNAs) showing increased expression in fine needle aspiration (FNA) samples at our institution. **(A)** LRRC52-AS1, **(B)** LINC02082, and **(C)** UNC5B-AS1. ***p < 0.001.

The expression of the candidate lncRNAs was examined in FNA samples to confirm their diagnostic value. To confirm the diagnostic ability of LRRC52-AS1, LINC02082, and UNC5B-AS1 for PTC, we evaluated the expression levels of the lncRNAs in samples collected from 51 patients who underwent FNA on thyroid nodules at our institution (**Figure 6**). Based on the Bethesda classification, 23 nodules were found to be benign (category [Cat] II), 10 were classified as Atypia of Undetermined Significance (AUS) (Cat III), and 18 were classified as Suspicious for Malignancy/Malignant (Cat V/VI). All nodules corresponding to Cat V/VI were surgically confirmed as PTC, except for that of one patient, who was lost to follow-up without surgery.

The characteristics of these patients are shown in **Table 3**. Their mean age was 50 years, and 52.9% of them were females; there was no difference between the three groups. The average tumor size was 1.81 cm, and the tumor size for the Cat II group was larger compared to the other groups.

**Table 3:** Characteristics of the patients who underwent fine needle aspiration (FNA) on thyroid nodules at our institution.

Parameters	Total (n = 51)	Category II (n = 23)	Category III (n = 10)	Categories V/VI (n = 18)
Age (years)	50.43 ± 12.95	49.65 ± 12.58	54.70 ± 16.34	49.06 ± 11.57
Sex				
Male	24 (47.1)	11 (47.8)	4 (40.0)	9 (50.0)
Female	27 (52.9)	12 (52.2)	6 (60.0)	9 (50.0)
Tumor size (cm)	1.81 ± 1.42	2.41 ± 1.58	1.46 ± 1.56^*^	1.22 ± 0.66^**^

^*^p < 0.05 and ^**^p < 0.01 vs. Category II.

When we compared Cat II and Cat V/VI, the expression levels of LRRC52-AS1, LINC02082, and UNC5B-AS1 were significantly higher in Cat V/VI than in Cat II. Based on the cut-off value of the highest Youden’s index, the sensitivity, specificity, negative predictive value (NPV), and positive predictive value (PPV) of each lncRNA was good, ranging from 83.3–88.9%, 91.3–100.0%, 88.5–91.3%, and 88.9–100.0%, respectively (**Table 4**). If two or more of the LRRC52-AS1, LINC02082, and UNC5B-AS1 lncRNAs are above the cut-off value, better results can be achieved, with 88.9% sensitivity, 100.0% specificity, NPV 92.0%, and PPV 100.0%, for PTC diagnosis using FNA samples.

**Table 4:** Diagnostic performance of long non-coding RNA (lncRNA) expression for the comparison of Bethesda II (n = 23) vs. Bethesda V/VI (n = 18) fine needle aspiration (FNA) samples at our institution.

Official symbol (cut-off value)	Sensitivity % (95% CI)	Specificity % (95% CI)	^a^NPV % (95% CI)	^b^PPV % (95% CI)
LRRC52-AS1 (4.5874)	83.3 (57.7–95.6)	100.0 (82.2–100.0)	88.5 (68.7–97.0)	100.0 (74.7–100.0)
LINC02082 (1.5193)	88.9 (63.9–98.1)	91.3 (70.5–98.5)	91.3 (70.5–98.5)	88.9 (63.9–98.1)
UNC5B-AS1 (2.5240)	88.9 (63.9–98.1)	91.3 (70.5–98.5)	91.3 (70.5–98.5)	88.9 (63.9–98.1)
One or more	100.0 (71.8–100.0)	82.6 (60.5–94.3)	100.0 (79.1–100.0)	81.8 (59.0–94.0)
Two or more	88.9 (63.9–98.1)	100.0 (82.2–100.0)	92.0 (72.5–98.6)	100.0 (75.9–100.0)
All three	72.2 (46.4–89.3)	100.0 (82.2–100.0)	82.1 (62.4–93.2)	100.0 (71.7–100.0)

^a^NPV, negative predictive value; ^b^PPV, positive predictive value; 95% CI, 95% confidence intervals.

Ten patients with a thyroid nodule classified as Cat III, after consideration of worrisome clinical and sonographic features, were followed up with sonography or a repeat of FNA according to the guidelines (**Table 5**) (1, 25). Two out of ten thyroid nodules, originally diagnosed as AUS *via* FNA, were re-classified as Cat V *via* a second FNA, and finally diagnosed as PTC based on postoperative pathology results. In these two thyroid nodules, two or more of the LRRC52-AS1, LINC02082, and UNC5B-AS1 lncRNAs were above the cut-off value. These results imply that a combination of lncRNA expression levels can be applied to the actual molecular diagnosis of PTC.

**Table 5:** Follow-up results of 10 patients with a thyroid nodule classified as Category III (0 = under the cut-off value, 1 = above the cut-off value).

Patient	LRRC52-AS1(4.5874)	LINC02082(1.5193)	UNC5B-AS1(2.5240)	SUM	Follow-up
1	0	1	1	2	Category V
2	0	0	0	0	Sono f/u
3	1	0	0	1	Category II
4	0	0	1	1	Sono f/u
5	0	0	0	0	Category II
6	1	1	1	3	Category V
7	1	0	1	2	Not yet
8	1	0	0	1	Not yet
9	0	0	1	1	Not yet
10	0	0	0	0	Category III

f/u, follow-up.

## Discussion

**Figure 7:**
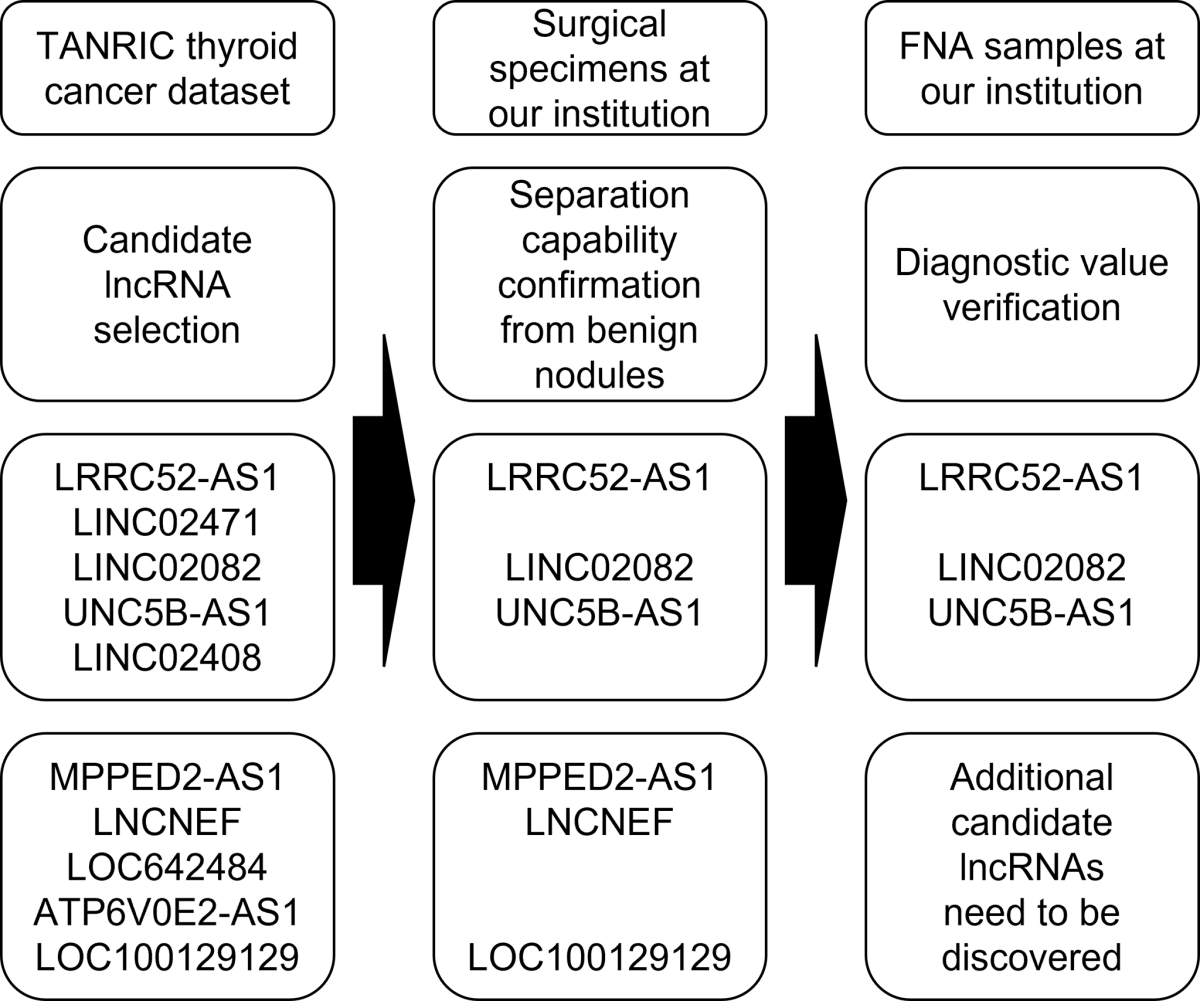
Flowchart of a study evaluating the diagnostic value of selected long non-coding RNAs (lncRNAs) for the molecular diagnosis of papillary thyroid cancer (PTC).

In this study, we evaluated the diagnostic value of selected lncRNAs for the molecular diagnosis of PTC (**Figure 7**). LncRNA expression analysis exhibited high diagnostic efficiency, and certain combinations of lncRNAs exhibited better results than single lncRNAs in the differential diagnosis of benign thyroid tumors and PTC.

First, we selected candidate lncRNAs with PTC diagnostic values by analyzing TANRIC thyroid cancer dataset, an open-access resource for interactive exploration of lncRNAs in cancer (22). In our previous study, we established that one of the candidates, LINC02082, had higher levels in almost all (60/64, 93.8%) PTC tissues than in the matched normal tissues (21). For the diagnosis of PTC, the differences in expression between malignant nodules and benign nodules are more important than those between malignant nodules and normal thyroid tissues. Therefore, to check and confirm the separation capability of candidate lncRNAs, we compared the expression levels of candidate lncRNAs in the malignant nodular tissues, benign nodular tissues, and paired normal thyroid tissues of a few patients who underwent thyroidectomy at our institution. The expression levels of LRRC52-AS1, LINC02082, UNC5B-AS1, MPPED2-AS1, LNCNEF, and LOC100129129 were significantly increased or decreased in malignant nodules compared to those in paired normal thyroid tissues and also in benign nodules.

Next, ROC curves were used to analyze the potential diagnostic efficacy of these lncRNAs in TANRIC thyroid cancer dataset, and these lncRNAs showed potential for diagnostic use. It was expected that the potential diagnostic efficacy could be further improved when using the modified expression level as calculated by dividing the increased lncRNA level by the decreased lncRNA level. When measuring the RNA expression levels for cancer diagnosis, a housekeeping gene control such as GAPDH, whose expression levels remain the same, is required (26). However, most lncRNAs have a low expression level, and there is no known lncRNA housekeeping gene; most housekeeping genes, such as GAPDH, have a relatively high expression level, which may be inappropriate for accurate lncRNA quantitative analysis (27). When dividing the increased lncRNA level by the decreased lncRNA level, the use of the housekeeping gene becomes unnecessary, and by maximizing the difference in the levels, it is possible to significantly increase the accuracy of cancer diagnosis.

We applied the data of these lncRNAs to FNA samples for verification. However, in the preliminary FNA test, the lncRNAs whose levels decreased in PTC did not exhibit pronounced diagnostic performance as expected. Moreover, due to the limited amount of FNA samples, we focused on the lncRNAs whose levels increased in PTC. The lncRNAs whose levels increased in PTC exhibited a favorable diagnostic performance, and two or more lncRNAs above the cut-off value showed an even better diagnostic performance. Additionally, in a cumbersome category, Cat III, these lncRNAs showed a remarkable diagnostic value. These data suggest that LRRC52-AS1, LINC02082, and UNC5B-AS1 can be used to differentiate between benign and malignant tumors in thyroid nodules by measuring their expression levels, which has not been reported in PTC thus far.

Interestingly, the expression levels of LRRC52-AS1, LINC02082, and UNC5B-AS1 were not elevated in a surgical specimen of non-invasive encapsulated follicular thyroid neoplasm with papillary-like nuclear feature (NIFTP), although the data were not included in the study. These lncRNAs are believed to aid in the diagnosis of borderline thyroid tumors (hyalinizing trabecular tumor, follicular tumor of uncertain malignant potential, well-differentiated tumor of uncertain malignant potential, and NIFTP) that are difficult to diagnose; however, additional research such as follicular neoplasm is needed (28).

Although the data from this study demonstrated the diagnostic value of lncRNAs, interestingly, limited information is available regarding the function of these lncRNAs. Zhou et al. showed that LRRC52-AS1 is associated with clinical progression and regulates cell migration and invasion in PTC (29). Our previous study demonstrated that LINC02082 expression is elevated in human thyroid cancer, and it may play a critical role in thyroid carcinogenesis (21). Wang et al. showed that the lncRNA UNC5B-AS1 promotes proliferation, migration, and invasion in PTC cell lines (30). There are also several studies showed that other many lncRNAs, such as MALAT1, H19, BANCR, HOTAIR, GAS5, and PCA3, play an important role in regulation of different processes involved in the development and progression of various thyroid cancers, and suggested these lncRNAs could be used as novel biomarkers for early diagnosis or even treatment (17, 31, 32). However, these previous studies only analyzed in thyroid cancer cell lines or only compared thyroid cancer tissues and adjacent normal thyroid tissues from surgical specimen. In the present study, we additionally compared the lncRNA data of these tissues with those of benign nodules and applied to actual FNA samples to show that these lncRNAs have a real diagnostic value. Although further research is needed, these lncRNAs are expected to be helpful in the prognosis as well as diagnosis of PTC, based on currently available reports (19, 33).

To date, studies on lncRNAs in thyroid cancer have been limited compared with studies on protein-coding RNAs (34). LncRNAs, such as BANCR, are relatively well-known; however, most have been only discovered recently (35). The functions of lncRNAs are dictated by their secondary structures rather than their primary sequences, and their subcellular localization is critical for their function (36, 37). Researchers may need to perform gene regulation *via* CRISPR activation, CRISPR inhibition, or antisense LNA GapmeRs rather than *via* plasmid cDNA, siRNA, or shRNA to study lncRNA functions (38–41). Animal experiments can be challenging because lncRNAs exhibit evolutionarily poor sequence conservation across species (17). The fact that lncRNAs are the functional units themselves may be more meaningful for diagnosis than measuring mRNA levels (37). However, the measurement of lncRNA levels is difficult, as the expression of lncRNAs is generally considerably lower than that of mRNAs (17). In this study, several representative lncRNAs were measured using the laboratory-level real-time PCR method; however, if more lncRNAs are measured using a more sophisticated method, lncRNAs could be used as effective markers for advancing the molecular diagnosis of PTC.

This study has several limitations. First, the number of nodules included in the study was small, and there were not many indeterminate nodules that were difficult to diagnose. Second, only PTC was included in the study since it is the most common type of thyroid cancer, while the other types of thyroid cancer were not included. Third, in the preliminary FNA test, the diagnostic performance with the decreased lncRNA levels in PTC was lower than expected, and we did not discover additional candidate lncRNAs to improve the diagnostic accuracy. Fourth, false positive or false negative results might exist for the cytologically proven nodules in the FNA samples, as not all patients were surgically confirmed. Nevertheless, we showed that lncRNA expression analysis could be a promising approach for advancing the molecular diagnosis of PTC.

In summary, we selected candidate lncRNAs from TANRIC thyroid cancer dataset, confirmed the diagnostic potential of lncRNAs through our surgical specimen analysis, and verified the diagnostic value of lncRNA by using FNA samples at our institution. We showed that more accurate results could be obtained using a combination of specific lncRNAs. With further research on the identification of lncRNAs with additional diagnostic value and verification in large-scale studies of indeterminate nodules that require actual molecular diagnosis, the diagnostic method using lncRNAs could potentially supplement or replace FNA cytology and other existing molecular tests.

## Data availability statement

The original contributions presented in the study are included in the article/supplementary material. Further inquiries can be directed to the corresponding author.

## Ethics statement

The studies involving human participants were reviewed and approved by the Institutional Review Board of Severance Hospital (No. 4-2019-0335). The patients/participants provided their written informed consent to participate in this study.

## Author contributions

DK: conceptualization, methodology, formal analysis, investigation, resources, data curation, writing - original draft, visualization, funding acquisition. JY: validation, formal analysis, investigation, data curation, writing - review and editing. JK: resources, writing - review and editing. Y-aH: resources, writing - review and editing. JKK: methodology, investigation, resources, writing - review and editing. CRK: methodology, resources, writing - review and editing. JHY: methodology, investigation, resources, writing - review and editing. JYK: methodology, investigation, resources, writing - review and editing. K-HN: conceptualization, methodology, investigation, resources, writing - review and editing, supervision, project administration, funding acquisition. EJL: conceptualization, writing - review and editing, supervision. All authors contributed to the article and approved the submitted version.

## Funding

This research was supported by the Basic Science Research Program through the National Research Foundation of Korea (NRF) funded by the Ministry of Education (2021R1I1A1A01044993) and the Severance Hospital Research fund for clinical excellence (SHRC) (C-2019-0016).

## Acknowledgments

The authors would like to thank Editage (www.editage.co.kr) for English language editing.

## Conflict of interest

The authors declare that the research was conducted in the absence of any commercial or financial relationships that could be construed as a potential conflict of interest.

## Publisher’s note

All claims expressed in this article are solely those of the authors and do not necessarily represent those of their affiliated organizations, or those of the publisher, the editors and the reviewers. Any product that may be evaluated in this article, or claim that may be made by its manufacturer, is not guaranteed or endorsed by the publisher.
